# Evaluation of Root Canal Morphology of Maxillary First Premolars by Cone Beam Computed Tomography in Saudi Arabian Southern Region Subpopulation: An In Vitro Study

**DOI:** 10.1155/2019/2063943

**Published:** 2019-02-27

**Authors:** Shadia Maghfuri, Husain Keylani, Hitesh Chohan, Salha Dakkam, Aeshah Atiah, Mohammed Mashyakhy

**Affiliations:** ^1^General Practitioner, Jazan, Saudi Arabia; ^2^Endodontist, Ministry of Health, Dental Center, Jazan, Saudi Arabia; ^3^Department of Restorative Dental Sciences, Endodontic Division, College of Dentistry, Jazan University, Jazan, Saudi Arabia

## Abstract

**Introduction:**

The aim of this study was to investigate the root canal morphology of maxillary first premolars in Saudi Arabian subpopulation by cone beam computed tomography (CBCT).

**Methods:**

One hundred maxillary first premolars were collected from the College of Dentistry at Jazan University and different dental centers in the southern region of Saudi Arabia. These teeth were cleaned thoroughly and were mounted in a plastic artificial mandible jaw to mimic soft tissue which was then placed in a water container. The 3D scan images were obtained with CBCT imaging (3D Accuitomo170) and evaluated on the basis of the number of roots, number of canals, and root canal configuration using Vertucci's classification.

**Results:**

Out of the 100 maxillary first premolars, the majority of teeth had 2 roots (61%) followed by (36%) one root. Two canals were the most predominant with 97% and then 3% three canals, and no tooth presented with one canal. Type IV canal configuration was most prevalent (75%) followed by Type V (13%).

**Conclusions:**

The CBCT provides enhanced and accurate information of root morphology, canal configuration, and its variations, thereby constituting an excellent alternative for radiographic diagnosis tool in the dental practice.

## 1. Introduction

Endodontic treatment is an essential element of comprehensive, quality dental care. Successful endodontic treatment depends on complete root canal cleansing and shaping, three-dimensional hermetic root canal system (RCS) obturation, and well-fitting coronal restorations with no leakage [1]. A comprehensive understanding of roots and canals of a tooth is necessary for successful endodontic therapy [2]. However, lack of thorough knowledge about teeth internal anatomy is one of the main reasons for endodontic treatment failure where the clinician might miss some canal systems uncleaned [3]. The anatomical variations of the RCS are crucial in endodontic treatment [4]. Therefore, to achieve perfect shaping and cleaning followed by proper obturation to increase the success rate of root canal treatment (RCT), the clinician should be aware of the complexity of RCS and its variations [3]. Among different populations and even in different individuals within the same population, root canal morphology varies greatly. Therefore, an accurate knowledge of root canal morphology and its anatomical variations is essential for a successful endodontic treatment [1]. Various classifications have described the RCS of human permanent teeth including the Weine classification, Vertucci classification, and Gulabivala classification; however, Vertucci classification is still the most famous one. [4–7] Vertucci [6] classified the root canal systems into eight types as follow:

- Type I (1-1): single canal runs from the orifice to apex.
- Type II (2-1): two canals arise from the pulp chamber which unite in its course into one.
- Type III (1-2-1): one canal arises from the pulp chamber and during its course, splits into two. These two canals again unite into one before exiting from the apex.
- Type IV (2-2): two canals run separately from the orifice to apex.
- Type V (1-2): one canal arises from the floor of the pulp chamber and during its course, divides into two.
- Type VI (2-1-2): two canals start from the pulp chamber, and during its course, they unite into one and then again divide into two before exiting from the root apex.
- Type VII (1-2-1-2): one canal leaves the pulp chamber which divides and again unite into one in its course and finally divides into two before exiting from the apex.
- Type VIII (3-3): three canals leave the pulp chamber and run independently towards the apex.

The maxillary first premolars are among the most difficult teeth to be treated endodontically due to their variation in number of roots, canal system, and various pulp cavity configurations. These variations can make it difficult to visualize the apical limits of the roots on conventional periapical radiography [2]. Various factors like ethnicity, age, gender, and study design contribute to the variations found in root canal studies. Thereby, endodontic treatment for a maxillary first premolar could be challenging [2, 8].

In a study conducted by Awawdeh et al. to investigate root canal morphology of maxillary first premolars in a Jordanian population, it was observed that, out of 600 maxillary first premolars, 30.8% has one root, 63.2% has two roots, and 5.2% has bifid roots [9]. Pécora et al. studied the external and internal anatomy of 240 extracted maxillary first premolars. The results revealed that a total of 55.8% of the teeth had a single root, 41.7% had two roots, and 2.5% had three roots. Considering all of the first premolars, 17.1% had one canal, 80.4% had two canals, and 2.5% had three canals [10].

Numerous studies have dealt with the evaluation of root canal morphology among different populations using various techniques, such as radiography, decalcification, sectioning, replication, and computerised-aided techniques [1]. Different methods have been utilized to investigate root canal anatomy, including in vivo and in vitro methods. The in vivo techniques include clinical evaluation during RCT, retrospective assessment of patient records, conventional radiographic evaluation, and advanced radiographic techniques such as cone beam computed tomography (CBCT), while the *in vitro* methods include canal staining and tooth clearing, root sectioning, microscopic examination, examination of conventional radiographs, and using three-dimensional modalities such as microcomputed tomography (*μ*-CT) [4].

Radiographic investigation is important in diagnosis and RCT planning [11]. Such knowledge can assist in localisation and negotiation of canals, as well as their subsequent management [1]. The most commonly used X-ray technique in dental clinics is two-dimensional (2-D) intraoral periapical radiograph (IOPA) [12]. Conventional radiographs are used for the management of endodontic problems yield limited information because of the 2D nature of images produced, geometric distortion, and anatomical noise. Therefore, variation of root and root canal morphology cannot be determined precisely without 3D images during endodontic therapy [13]. To understand the complexity of teeth morphology, variation in the horizontal angle, i.e., 20° and 40°, improves the visualisation of additional (superimposed) canals in premolars [14]. However, in some maxillary teeth, it is impossible to do so because of shallow palatal vault [15].

CBCT is a noninvasive method, potentially provides the clinician with the ability to evaluate the maxillofacial anatomy in axial, sagittal, and coronal sections, and produces high quality 3D diagnostic images without structure overlapping [11]. CBCT imaging is a three-dimensional (3-D) X-ray modality that shows teeth and adjacent structures without superimposition [1]. CBCT scanning provides comprehensive information about the root canal morphology of maxillary premolar teeth [16]. Root and canal morphology, the number of canals, and their divergence or convergence from each other can be visualised in 3D [11]. These data may help clinicians in root canal treatment of premolar teeth [15]. For these reasons, CBCT has been recommended for the accurate evaluation of RCS [11]. CBCT improves the diagnosis capacity in dentistry, such as increased radiation dose to the patient and presence of artifacts on the image. In a study conducted by Bernardes et al., the images obtained on conventional periapical radiographs and 3D scans were compared for the diagnosis of root fractures. A statistical difference was observed in the results obtained from CBCT and conventional radiographs in the diagnosis of root fractures. Thus, the study concluded that CBCT was better than conventional radiography in the diagnosis of root fractures, thereby constituting an excellent alternative for diagnosis in general dental practice. CBCT provides achievement of 3D images, thereby allowing better conclusive diagnosis [16]. Neelakantan et al. reported that CBCT can examine the RCS as accurate as staining and clearing technique and it is more accurate than IOPA. Few literatures are available on root canal anatomy and its variation in maxillary first premolar in Saudi Arabian population [17–19]. So, the aim of this study was to investigate the root canal anatomy and morphology of maxillary first premolars by CBCT in a subpopulation in the southern part of Saudi Arabia.

## 2. Materials and Methods

In this study, the samples were collected from the College of Dentistry at Jazan University and some dental centers of the southern region of Saudi population. The process of collection was performed by a team of clinicians who were made to understand the aim of the study and every tooth was collected by a case record, stating and confirming the ethnicity of the patient. A total of 100 maxillary first premolars with mature and intact external morphology were included in the study.

**Figure 1:**
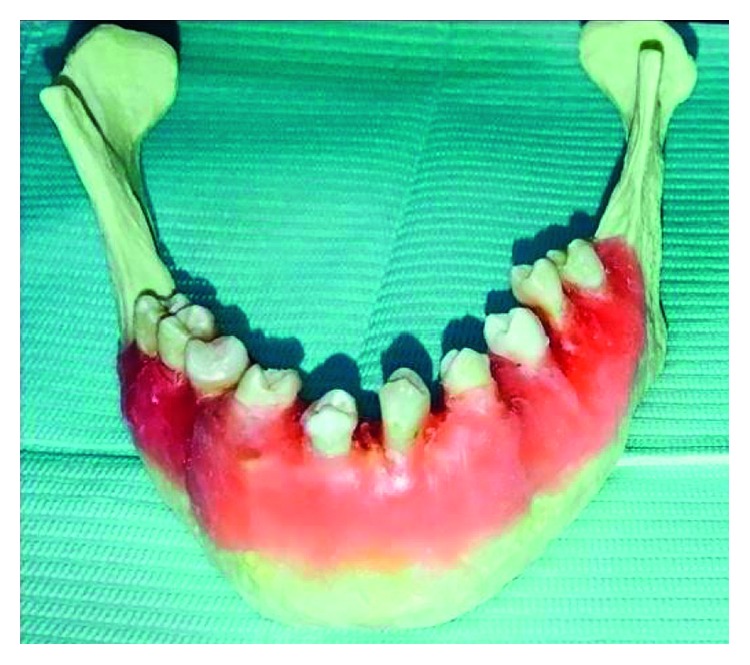
Teeth arrangement on plastic artificial jaw (*Note.* Color image).

The extracted teeth were thoroughly washed under tap water and immersed in 2.5% sodium hypochlorite to remove all soft tissue. The extracted teeth were mounted in a plastic artificial mandible jaw to mimic soft tissue which was then placed in a water container (Figure 1).

**Figure 2:**
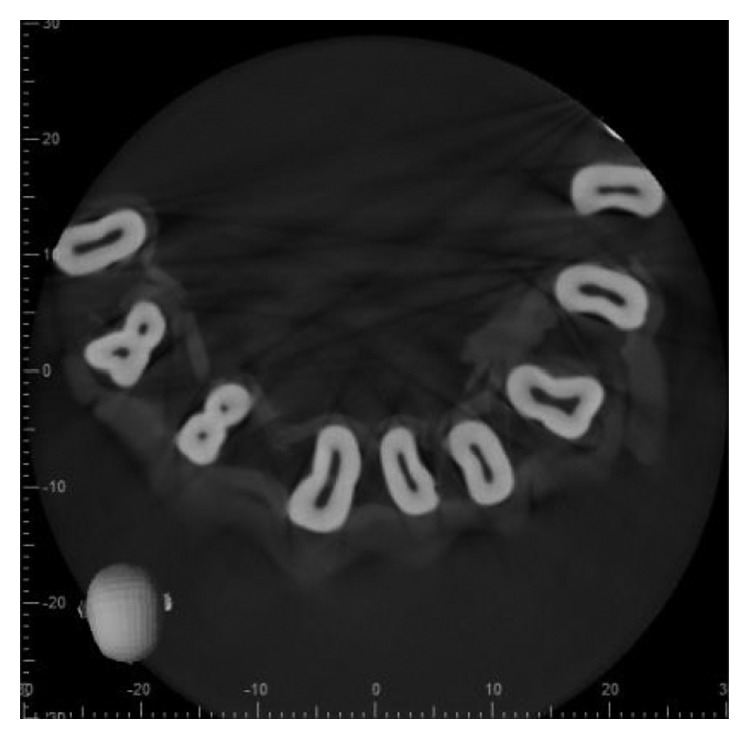
Axial section at cervical third (*Note*. Black and white image).

**Figure 3:**
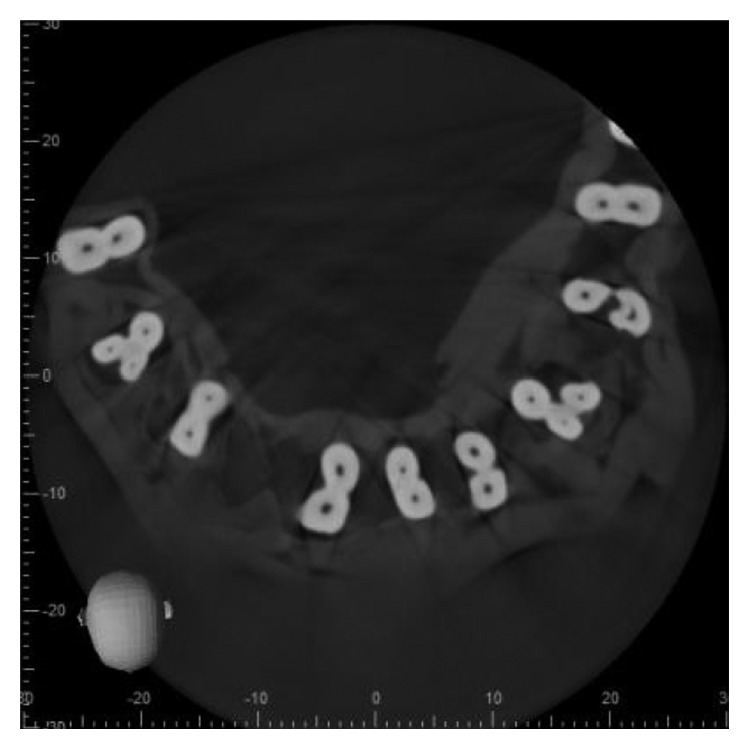
Axial section at middle third (*Note.* Black and white image).

**Figure 4:**
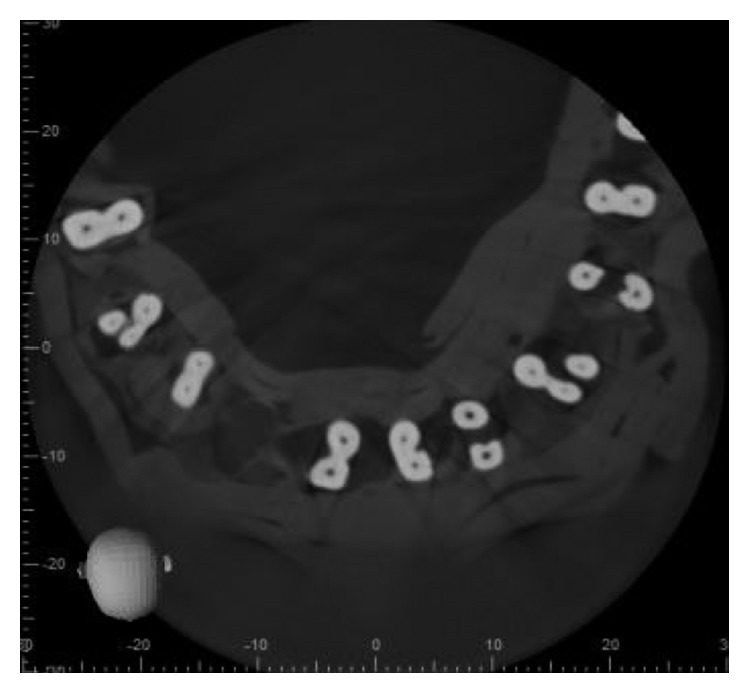
Axial section at apical third (*Note*. Black and white image).

The 3D scan images were obtained with CBCT imaging (3D Accuitomo170) set at 90 kV and 7.0 mA with an exposure time of 30.8 sec. The voxel size was 12,500 *μ*m, and the slice thickness was 0.250 mm. All the scans were performed by an experienced oral radiologist according to the manufacturer-recommended protocol necessary for adequate image quality. The images were then evaluated on the basis of the number of roots, canals, and root canal configuration using Vertucci's classification by one radiologist, two endodontists, and one general practitioner using I-Dixel imaging software in axial, coronal, and sagittal planes (Figures 2–4).

## 3. Results

**Figure 5:**
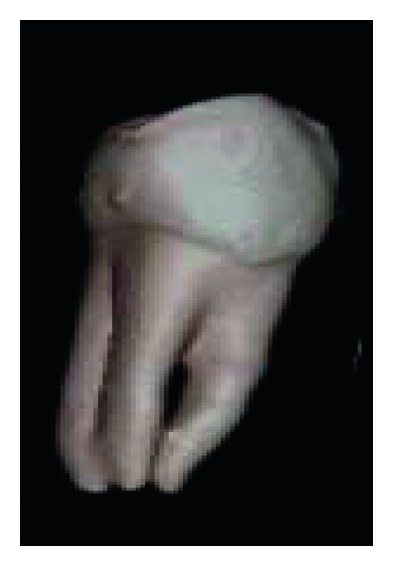
Three roots (*Note*. Color image).

**Figure 6:**
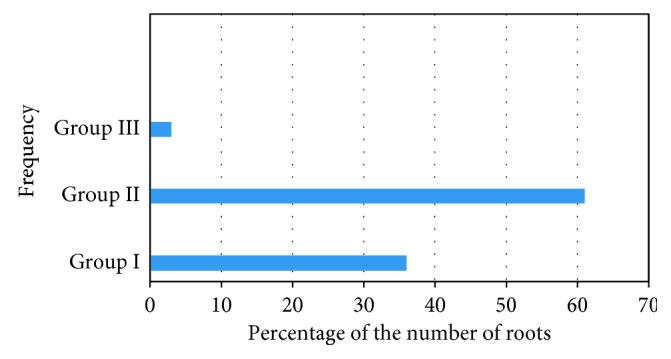
The frequency distribution and percentage of the number of roots in maxillary first premolars (*Note*. Color image).

According to the number of roots, the teeth were divided into three groups: group I (one-root form), group II (two-root form), and group III (three-root form). Out of 100 maxillary first premolars, 36 (36%) teeth had a single root, 61 (61%) teeth had two roots, and 3 (3%) teeth had three roots (Figures 5 and 6 and Table 1).

**Figure 7:**
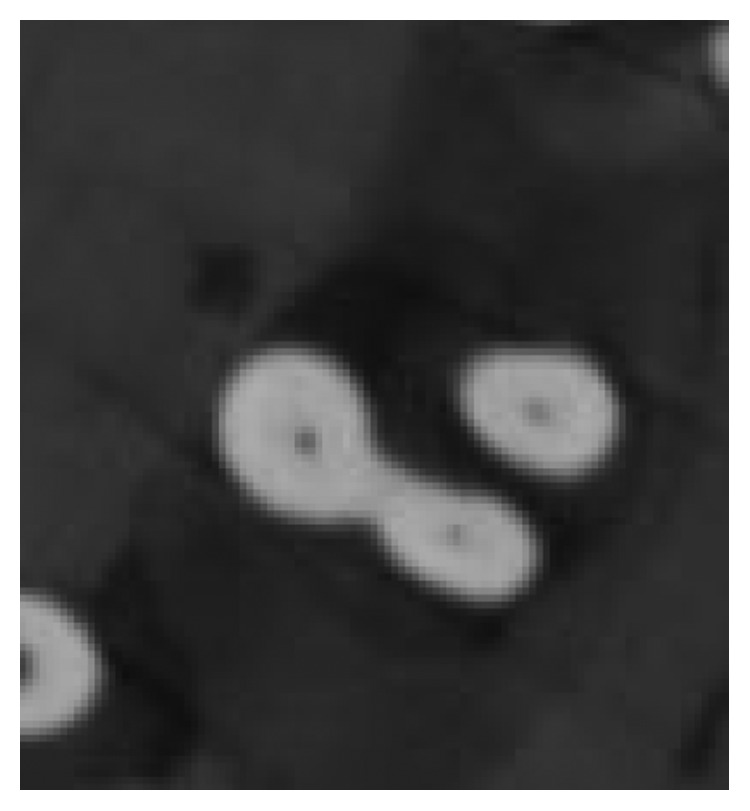
Type VIII (*Note*. Black and white image).

**Figure 8:**
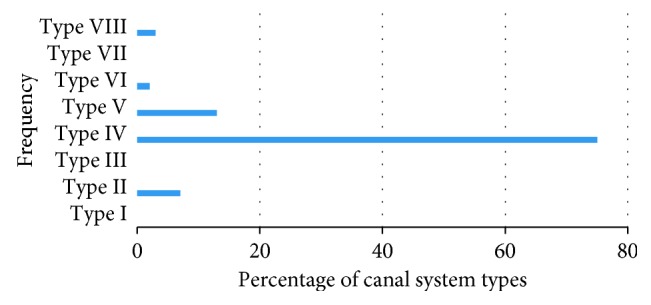
The frequency and percentage of canal system types according to Vertucci's classification in maxillary first permanent premolars *(Note*. Color image).

Two canals were the most predominant with 97% and then 3% three canals, and no tooth presents with one canal. On examination of the internal root anatomy, it was observed that Type IV canal configuration was most prevalent (75%), followed by Type V (13%), Type II (7%), and Type VIII (3%), while 2% exhibited Type VI configuration (Figures 7 and 8 and Table 2).

## 4. Discussion

Clear understanding of roots anatomy and canals morphology is paramount to perform efficient biomechanical cleaning and shaping for predictable endodontic outcome. Nevertheless, the variation of the root canal morphology presents clinical difficulties that might lead to unfavorable endodontic treatment [1, 2].

CBCT is an excellent ex vivo and in vivo method to evaluate external and internal root morphology compared to conventional 2D radiography [17]. Many studies have used CBCT methodology to evaluate external and internal anatomy of maxillary premolars [4, 11, 16, 21–23].

In the present ex vivo study, we evaluated 100 extracted first maxillary premolars by means of CBCT. The most commonly observed root morphology was two roots (61%), followed by one root (36%) and 3 roots (3%). In a recent study in Saudi population using in vivo CBCT with bigger sample size, almost similar findings were observed, where two roots were 75.1%, followed by single root (23.7%) and three roots (1.2%) [4]. In another study in the same population using visual radiography, digital radiography, and sectioning methods, in maxillary first premolars, the prevalence of two roots was 80.9%, followed by one root 17.9% and three roots 1.2% [20]. This study showed same findings compared to our results, regardless of the methodology.

In addition, we noted a higher prevalence of two-rooted maxillary first premolar in our study compared to Yemeni (44.4%), Turkish Cypriot (44.8%), and Spanish population (51.4%) [1, 15, 21]. The prevalence of three-rooted maxillary first premolars in our study (3%) was higher compared to Indian (0.4%) population, Yemeni population (0.8%), Turkish Cypriot population (0.9%), and German one (1.2%) [1, 2, 15, 22]. Also, we observed the low prevalence of single-rooted maxillary first premolars (36%) compared to Yemeni populations (54.8%), North Indian population (53.6%), and Chinese subpopulation (66%) [1, 2, 23].

All of the specimens in the present study corresponded to Vertucci's classification [6]. Type IV canal configuration was the most prevalent (75%), which is slightly higher than other studies done in the same population—Saudi Arabian (69.1%) [4] and (63%) [20]. It is also higher compared to other studies from different ethnical backgrounds: 55.6% in Yemen [1], 33.2% in India [2], 59.5% in Turkish Cypriot population [5], and 51% in Chinese subpopulation [23].

Identification, preparation, and obturation of Type IV and Type VIII canal configurations are relatively straightforward. In contrary, Types III, V, VI, and VII where the canal further divides within the root need extra armamentarium and expertise. The diagnostic and surgical aids like CBCT and surgical operating microscope are useful to appreciate such complex root canal system.

Regardless of the methodology and low number of the sample size in the present study, the results are consistent with the other two studies done in the same population using different methodologies with slight variations.

## 5. Conclusion

The CBCT provides enhanced and accurate information of root morphology, canal configuration, and its variations, thereby constituting an excellent alternative for diagnosis in the dental practice. Within the limitation of the present study, it showed a high incidence of two-rooted, two-canal maxillary first premolars, and Type IV Vertucci's configuration. CBCT scanning provides comprehensive information about the root canal morphology of maxillary premolar teeth and indicated with a small field of view once IOPA cannot provide enough diagnostic information. Further study should be conducted using higher numbers for more reliable results.

## Figures and Tables

**Table 1:** The frequency distribution and percentage of the number of roots in maxillary first premolars.

Group	No of roots	No. of teeth (*n*%)
Group I	One	36 (36%)
Group II	Two	61 (61%)
Group III	Three	3 (3%)

**Table 2:** The frequency and percentage of canal system types according to Vertucci's classification in maxillary first permanent premolars.

Canal type	No. of teeth (*n*%)
Type I	—
Type II	7
Type III	—
Type IV	75
Type V	13
Type VI	2
Type VII	—
Type VIII	3

## Data Availability

The data used to support the findings of this study are included within the article.
